# Medical Society of Nashville

**Published:** 1843-07

**Authors:** 


MEDICAL SOCIETY OF NASHVILLE.

The physicians of Nashville have commenced a spirited organization under the above title, for the cultivation of medical science and the improvement of its members, as well as the establishment of a code of professional ethics. The Society has held several meetings, elected officers, and made altogether an auspicious beginning. Nearly all the physicians of Nashville are members. The officers of the Society are Boyd McNairy, M.D., President; G. W. Dickinson, M.D., Vice President; A. H. Buchanan, M.D., Corresponding Secretary; R. C. K. Martin, M.D., Recording Secretary; and C. K. Winston, M.D., Treasurer. The officers are elected for one year.

The discussions that have taken place at the meetings of the Society displayed a zeal in the cause of medical learning, and a spirit of research highly creditable to the members, and which augurs well for its good influence upon the profession. We hope this association will lead to the formation of similar societies in all the towns of the State. *E collisione scintilla*, is a proposition as true of mind as of the flint and steel. It is professional intercourse that we want—the collision of mind with mind—the comparing of experience and observation—the excitement and the ardor which are enkindled by the meeting of generous spirits engaged in the same high pursuits. The benefit that would flow from such associations continued for a generation, and conducted with liberality and spirit, is incalculable. We have only to look abroad to Paris, London, Dublin, Edinburgh, and to Philadelphia, and New York, in our own country, to see the benign effects of large professional intercourse, and generous professional rivalry. These associations are the nuclei around which scientific discoveries are collected—the foci of intellect, imparting an influence that extends over the world. Y.

